# 

**Published:** 1914-02-14

**Authors:** 


### CONTENTS.

| | PAGE |
|---|---|
| **Sanatoria Treatment and the Poor Law** ... | 521 |
| **The Bradford Royal Infirmary Project** ... | 522 |
| **Surgical Treatment of Diseases of Metabolism** | 529 |
| **The Need for Specialism** ... ... ... ... By M. O. | 530 |
| **A German School of Recovery** ... ... ... The Example of the Mannheim Municipality. | 531 |
| **The Poor-Law Institutions Order** ... ... The Duties of Workhouse Medical Officers. | 533 |
| **Reports on Hospitals of the United Kingdom—Series III.** ... ... ... ... ... ... ... By Sir HENRY BURDETT, K.C.B., K.C.V.O. The Northampton General Hospital. | 535 |
| **Hospital and Institutional News** ... ... ... ... New Scottish Lunacy Commissioners—A Radium Adventure at Liverpool—New Secretary of Bristol General Hospital—Should Hospital Street Collecting be Stopped?—Medical Evidence in the Liverpool Murder Case—Thirty Years as Workhouse Medical Officer—Pioneers of Infirmary Reform—Infirmary Conditions Thirty Years Ago—Farm Colony for the Feeble-Minded of Somerset—Wimbledon Isolation Hospital: An Inquiry—From Hospital Secretary to President—A Cheeseparing Policy at North Evington Infirmary—The Coroner and the Spirit of Parsimony—A Hospital and a Bull-Fight—Retirement of Sir Laurence Gomme—An Architect on the Open Basement—The Neglect of Voluntary Aid Detachments—Financial Crisis at the Polyclinic—Death of a Hospital Founder—The Blackpool Hospital Deadlock—The Palladium Matinée—The Registration of Nurses—Salvarsan Ward for the London Hospital—Death of Miss Julia Cock—Institutional Pharmacists and the Pharmaceutical Society—This Week's Drug Market. | 523 |

### CONTENTS—(*continued*).

PAGE

**The Origin and Evolution of the 18th Century Hospital Movement** ... ... ... ... 538

**National Insurance Act Developments** ... ... 539

Penalties for Non-Payment of Arrears.

**Welsh Sanatorium Construction and Equipment** ... ... ... ... ... ... ... ... 541

A Discussion at the Sanitary Institute.

**The Bradford Infirmary Project** ... ... ... 542

**Round the World** ... ... ... ... ... ... 543

Fellow-Workers and Their Doings.

**The Editor's Letter-Box** ... ... ... ... ... 544

National Medical Union.

Mr. George's Panel Statistics.

South Shields Guardians and the Use of Alcohol.

Examination Questions for Red Cross Students.

The Altheat Stove.

The Elimination of the Middle Class.

The Control of the Invalid Children's Aid Association.

PAGE

**A Test Case at Torquay** ... ... ... ... ... 545

**Doctors' Wills** ... ... ... ... ... ... ... 545

**The Profession of Medicine** ... ... ... ... 546
The Organisation of the Profession in Ireland.
The National Medical Union.

**The Institutional Worker** ... ... (Suppl.) 1
Ward Furniture.
Questions Invited.

**Our Bureau of Information** ... ... ,, 2
Acknowledgments.
Questions for February.
The Sick and in Need.
The Housekeepers' Department.
Employment and Training.
Miscellaneous.

**Appointments** ... ... ... ,, 3

**London County and Westminster Bank** ,, 3

